# Epigenetic and genomic profiling of chordoid meningioma: implications for clinical management

**DOI:** 10.1186/s40478-022-01362-3

**Published:** 2022-04-19

**Authors:** Elena V. Daoud, Kelsey Zhu, Bruce Mickey, Hussein Mohamed, Mandisa Wen, Michael Delorenzo, Ivy Tran, Jonathan Serrano, Kimmo J. Hatanpaa, Jack M. Raisanen, Matija Snuderl, Chunyu Cai

**Affiliations:** 1 Department of Pathology, UT Southwestern Medical Center, 5323 Harry Hines Blvd, H2.130, Dallas, TX 75390-9073 USA; 2 Department of Pathology, NYU Langone Medical Center, New York, NY USA; 3 Department of Neurological Surgery, UT Southwestern Medical Center, Dallas, TX USA

**Keywords:** Chordoid meningioma, Global methylation profile, WHO grade, Prognosis, Atypical meningioma, Brain tumor

## Abstract

Chordoid meningioma is a morphological variant of meningioma designated as WHO grade 2. However, the recurrence rates varied widely in different case series, and to date, a unifying molecular genetic signature has not been identified. Among 1897 meningiomas resected at our institution, we identified 12 primary chordoid meningiomas from 12 patients. Histologically, all 12 cases had predominant (> 50%) chordoid morphology. Ten were otherwise grade 1, and two were also atypical. We performed DNA global methylation profile, copy number variation analysis, and targeted next-generation sequencing on 11 chordoid meningiomas, and compared to those of 51 non-chordoid, mostly high grade meningiomas. The chordoid meningiomas demonstrated a unique methylation profile in tSNE, UMAP, and hierarchical heatmap clustering analyses of the most differentially methylated CpGs. The most common copy number variation in chordoid meningioma was loss of 1p (7/11, 64%). Three chordoid meningiomas had 2p loss, which was significantly higher than the non-chordoid control cohort (27% vs 7.2%, *p* = 0.035). 22q loss was only seen in the two cases with additional atypical histological features. Chordoid meningiomas were enriched in mutations in chromatin remodeling genes *EP400* (8/11,73%) *KMT2C* (4/11, 36%) and *KMT2D* (4/11, 36%), and showed low or absent *NF2, TERT, SMO*, and *AKT1* mutations. Prognosis wise, only one case recurred. This case had atypical histology and high-grade molecular features including truncating *NF2* mutation, 1p, 8p, 10, 14, 22q loss, and homozygous deletion of *CDKN2A/B*. Progression free survival of chordoid, otherwise grade 1 meningioma was comparable to non-chordoid WHO grade 1 meningioma (*p* = 0.75), and significantly better than chordoid WHO grade 2 meningioma (*p* = 0.019). Conclusion: the chordoid histology alone may not justify a universal WHO grade 2 designation. Screening for additional atypical histological or molecular genetic features is recommended.

**Supplementary Information:**

The online version contains supplementary material available at 10.1186/s40478-022-01362-3.

## Introduction

Chordoid meningioma is a rare meningioma variant characterized by cords of eosinophilic, often vacuolated cells in an abundant mucoid matrix [19]. While descriptions of “myxoid”, “chondroblastic”, and meningioma mimicking a chordoma have been noted since 1970s [5, 7, 11, 14], the first time these tumors were categorized and termed as chordoid meningiomas was in 1988 [15]. In this initial series, the tumors showed prominent peritumoral lymphoplasmacytic inflammation that resembled Castleman’s tumors (angiofollicular hyperplasia of lymph nodes), and occurred in young individuals with other systemic manifestations of Castleman syndrome, including microcytic anemia, polyclonal gammopathy, or hepatosplenomegaly, most of which resolved after the tumor removal. Only two of the seven cases recurred. However, subsequent case series found that chordoid meningiomas predominantly occurred in adults in supratentorial locations, and most had no associated systemic hematopoietic abnormalities. The tumor recurrence rates varied widely in different series and ranged from 0 to 53% [1, 24]. Chordoid meningioma has been designated WHO grade 2 in the previous and current editions of WHO central nervous system tumor classifications. However, many patients had long progression free survival, and the majority of the tumor recurrences could be attributed to either incomplete resections or presence of other atypical features [4, 6, 13, 24, 32, 33]. In a meta-analysis of 221 chordoid meningiomas [1], the extent of chordoid component was not predictive of recurrence. Rather, the extent of resection and the Ki67 proliferation index (MIB) > 5% were the only factors that significantly associated with recurrences. As such, it is debatable whether the chordoid histology in itself portends a higher risk for recurrence.

Several recent studies examined the genetic and epigenetic profiles of chordoid meningiomas. Sievers et al. performed global DNA methylation analysis on 38 chordoid meningiomas[28], of which 24 fell into benign methylation classes (MC), 13 into intermediate MC, and one into malignant MC. Recurrences was predominantly seen in tumors with intermediate or malignant MC, only 1 in 20 tumors with the benign designation and long-term follow up recurred. Sugur et al. [29] performed fluorescence in situ hybridization (FISH) analysis on 15 chordoid meningiomas and found that recurrent chordoid meningiomas had a high rate of 22q, 18p, 14q, and 1p loss, similar to non-chordoid high grade meningiomas, while non-recurrent chordoid meningiomas showed few such deletions. Georgescu et al. [9] performed paneled NGS on 31 chordoid meningiomas and whole exome sequencing on 15 chordoid meningiomas, and identified 3 prognostic groups. The group with highest recurrence rate (ED2) was highly enriched in mutations of *NF2*, a gene whose loss of function is highly associated with atypical meningioma [10]. The group with moderate recurrence rate (ED3) had mutually exclusive *TRAF7, KLF4*, *AKT1* or *VHL* mutations. The group with no recurrence (ED1) had no mutations. Taken together, it seems that chordoid meningiomas are comprised of a molecularly heterogeneous group of tumors. The aggressiveness of chordoid meningioma appears to be associated with the same combinations of genetic and epigenetic alterations seen in conventional atypical meningiomas.

In this study, we provide detailed clinical history, histological assessment, DNA global methylation profile, copy number variation analysis, and targeted next-generation sequencing on 12 primary chordoid meningiomas in adults. We report a unique clustering pattern for chordoid meningiomas on methylation profile, separate from 51 conventional, mostly high grade meningiomas. Recurrence in chordoid meningioma was only seen in cases with concomitant atypical histology and molecular features.

## Materials and methods

### Case selection and histological assessment

Retrospective review of all meningiomas resected between 1995 and 2018 at the University of Texas Southwestern Medical Center identified 15 primary chordoid meningiomas from 15 patients, which accounted for 0.79% (15/1897) of all meningiomas. All chordoid meningioma slides were reviewed. The extent of chordoid composition was calculated on all slides of chordoid meningioma specimens. Mitotic index and presence or absence of brain invasion, necrosis, small cell change, hyper-cellularity, sheeted architecture, and macro-nucleoli were reviewed according to the 2016 WHO classification of central nervous system tumors. Analysis of tissue and clinical data was performed in accordance with local ethical regulations and approved by the institutional review board (IRB).

### Statistical analysis

Only patients with more than a year of post-operative brain MRI follow up were included in the survival analyses. These include 13 chordoid meningiomas from 12 patients and 464 cases of non-chordoid meningiomas. The latter included 332 grade 1 meningiomas, and 128 grade 2 meningiomas without any chordoid morphology. Clinical data, which included age, gender, race, presenting symptoms, tumor location, treatment, extent of resection, and radiographic follow-up, were obtained from the medical records. Statistical analyses were performed using GraphPad Prism version 9.1.0 software (GraphPad, La Jolla, CA) or SAS version 9.2 (SAS Institute Inc, Cary, North Carolina). Progression free survival (PFS) and overall survival (OS) analyses were performed using the Kaplan–Meier method. Risk factors for recurrence and hazard ratios were calculated by univariate Cox regression analysis. The Chi-square test or Fisher’s exact test were used to compare categorical parameters. The Wilcoxon–Mann–Whitney test was used to compare the age distribution. A two-sided probability level of 0.05 was chosen for statistical significance.

### DNA extraction, DNA methylation analysis and targeted next-generation sequencing

Global DNA methylation analysis and targeted next-generation sequencing (NGS) were performed on a total of 64 cases, including 11 primary chordoid and 53 non-chordoid meningiomas. The latter group was enriched in aggressive meningiomas, including 41 atypical meningiomas, 4 anaplastic meningiomas, 4 recurrent WHO grade 1 meningiomas, and 4 non-recurrent WHO grade 1 meningiomas. Two atypical meningiomas with poor methylation data quality were excluded from subsequent analysis. For chordoid meningiomas, blocks with the highest percent of chordoid morphology were selected for methylation analysis.

### Whole genome methylation profiling

DNA was extracted from formalin-fixed paraffin-embedded (FFPE) meningioma samples using Maxwell Promega FFPE kit (Promega Inc, WI). DNA methylation profiling was performed as described previously [27]. The samples were scanned in 8 batches with a mixture of Infinium type I and II probes. The raw signal intensity data from the Human Methylation Epic arrays were then processed in R. The variability in methylation across the physical slide was identified by Singular Value Decomposition (SVD) analysis on 64 samples. Beta-mixture quantile dilation (BMIQ) normalization on Infinium I and II probes with a 0.01 cutoff threshold was then performed and followed by a ComBat batchbeadchip adjustment on the physical slide. XY chromosome linked probes were also filtered out. The differential methylation analysis was performed using the ChAMP R package (version v2.16.2). DNA methylation data were then reduced to 2-dimension by distributed stochastic neighbor embedding (t-SNE) [31] and Uniform Manifold Approximation and Projection (UMAP) for visualization [20]. An optimal perplexity of 5 was chosen for both t-SNE and UMAP. The top 5000 most variable differentially methylated probes (DMP) were used for hierarchical clustering. Euclidean metric was used to measure distances in high dimension space.

### Targeted next-generation sequencing

Mutational and copy number analysis was performed on FFPE extracted DNA as described above and sequenced using clinically validated DNA next-generation sequencing exonic NYU Langone Genome PACT, a custom-designed Next-Generation Sequencing (NGS) panel targeting all exons and selected promoters of 580 cancer associated genes. The DNA libraries were hybridized with capture probes from IDT (Coralville, Iowa), and sequenced on Illumina NextSeq500. Raw sequence reads were trimmed and adapter sequences were removed before being mapped to the human genome hg19 using the BWA mem aligner (version 0.7.17) [17].

As matched DNA samples from patients were not available, somatic variants were called by mutect2 of GATK4 [2] on tumor samples with the pool of normal reference samples created from 54 blood samples from healthy subjects. Variants were then annotated by Ensembl Variant Effect Predictor (version 101) [21]. Sequencing artifacts were removed before post filtering was applied (the read coverage greater than 100x and tumor variant allele frequency greater than 10 % and less than 70%) to select highly confident calls. Copy number segments were calculated by Circular Binary Segmentation (CBS) and adjusted for the tumor purity and single nucleotide polymorphism (SNP) allele counts using CNVKit (version 0.9.7) [30]. The GISTIC2 (version 2.0) [22] algorithm was used to identify regions with significant copy number aberrations from the Meningioma cohort.

## Results

### Demographics of patients with chordoid meningioma

Patient demographics are summarized in Table 1. Among the 12 patients with chordoid meningiomas, 9 were women and 3 were men, with a mean age of onset 46.9 years (range 34–66). Among the 464 patients with non-chordoid meningiomas resected during the same time period, 314 were women and 150 were men, with mean age 55.5 (range 16–88). While the gender distribution was similar among both groups (*p* = 0.76, Fisher's exact test), the patients with chordoid meningiomas were significantly younger than those with non-chordoid meningiomas (*p* = 0.0321, unpaired t-test).

**Table 1:** Demographic, Histological and Methylation Characteristics of Chordoid Meningioma

Patient-tumor	Age/Sex	Anatomic location	Percent chordoid	WHO grade	Mits/10HPF	MIB %	EOR	PFS (mo)	Recur	MC	CNV
1	42/M	Frontal convexity	100	1	0	7.5	NA	125	No	NA	NA
2-ut33	42/F	Frontal convexity	90	1	0	5.0	GTR	232	No	Inter-A	1p loss, 3q gain
3-ut34	46/F	Frontal falx	70	1	0	0.8	GTR	221	No	Ben-2	1p,3p,3q loss
4-ut36	46/F	Frontal convexity	100	1	0	0.3	GTR	170	No	Ben-2	1p,2p,2q loss
5-ut37	34/F	Sphenoid ridge	90	1	0	6.4	GTR	66	No	Ben-2	No loss/gain
6-ut38	54/F	Foramen magnum	60	1	0	3.0	GTR	31	No	Ben-2	No loss/gain
7-ut39	40/F	Frontal convexity	90	1	0	2.0	GTR	124	No	Ben-2	No loss/gain
8-ut40	37/F	Temporal tentorium	100	1	0	0.5	GTR	110	No	Ben-2	1p,2p loss
9-ut41	66/M	Foramen magnum	90	1	0	1.0	GTR	47	No	Ben-2	1p loss
10-ut42	45/F	Sphenoid ridge	80	1	0	5.8	STR	65	No	Ben-2	1p loss
11-ut35	61/M	Parietal falx	70	2	12	14.5	GTR	30	Yes (DOD)	Mal	1,8p,10,14,22q loss*
12-ut43	50/F	Temporal tentorium	80	2	5	9.2	GTR	38	No	Ben-2	1p,2p,22q loss

*Mits* mitoses, *HPF* high power fields, *EOR* extent of resection, *GTR* gross total resection, *STR* subtotal resection, *PFS (mo)* progression-free survival in months, *DOD* died of disease, *MC* methylation class, *CNV* copy number variation

^*^This tumor also shows homozygous CDKN2A/B deletions

Anatomically 10 cases were supratentorial and 2 were infratentorial; none occurred in the spinal cord. The most common locations were frontal convexities (4), para-falx or tentorium (4), and sphenoid ridge (3). No patient had a history of Castleman disease. Patient 6 had a history of mediastinal non-Hodgkin lymphoma, status post mediastinal radiation 9 years prior to meningioma presentation. No patient had prior intracranial radiation history. No family history of meningiomas were noted in any of the patients. Only one patient (Patient 7) had multiple meningiomas at presentation, a left tentorium chordoid meningioma and right optic nerve meningothelial meningioma without chordoid morphology, resected at two different time points. Three other patients (Patients 2, 4 and 10) developed new meningiomas distant from the resection site on surveillance brain MRI. Patient 4 was treated with gamma knife and patients 2 and 10 were followed.

### Clinical course, histology, and survival analysis

All except one chordoid meningioma had gross total resections and did not receive post-operative radiation therapy. One patient (patient 10) had extensive tumor at presentation, centered in the right sphenoid wing with encasement of the right carotid artery and extensions into the right optic canal, cavernous sinus, sphenoid sinus, nasal pharynx and orbit. A subtotal resection was performed to debulk the mass and decompress the optic nerve, followed by post-operative fractionated radiation (30 fractions /54 Gray) to residual tumor.

**Fig. 1:**
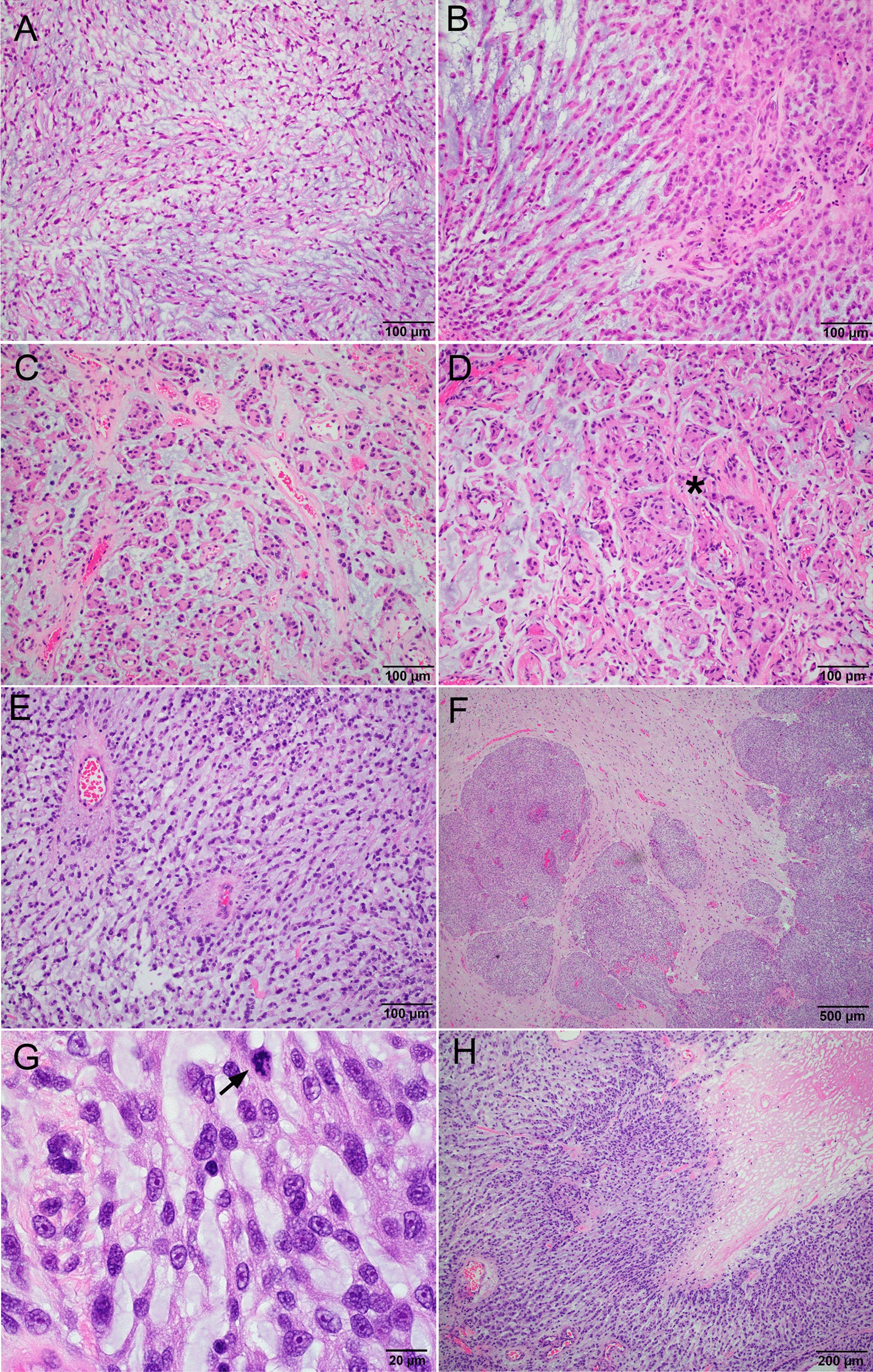
Chordoid meningioma histology. **A**–**D** Chordoid meningiomas with otherwise benign histology, H&E. **A** Case 4, tumor cells arranged as individual cells. **B** Patient 1, tumor cells arranged as single file cords. **C**,**D** Patient 6, tumor cells arranged as small nests. Asterisk indicates solid area without mucin. **E**–**H** Patient 11, Chordoid meningioma with atypical histology, H&E. **E** Chordoid morphology dominated the tumor. **F** Extensive brain invasion. **G** Mitosis (arrow) and prominent nucleoli. **H** Geographic necrosis

Histologically, the extent of chordoid component ranged from 60 to 100%. Only one tumor (patient 1) had extensive peritumoral lymphoplasmacytic infiltrate. Ten tumors, including all three tumors with pure chordoid morphology, were classified as otherwise WHO Grade 1 and lacked any atypical features; mitotic figures were rare (< 1/mm^2^) and there was no brain invasion. Tumor cells were arranged as individual cells (Fig. 1A), single file cords (Fig. 1B), or small nests (Fig. 1C) within abundant mucoid matrix. More compact areas with mucin depletion were indistinguishable from conventional meningothelial or transitional meningioma (Fig. 1D, asterisks) and were deemed “non-chordoid” areas. None of those tumors recurred after a mean brain MRI follow up of 110 months (range 31–232), including the case with subtotal resection.

Two cases (Patients 11 and 12) were histologically classified as atypical meningioma, WHO grade 2. Case 11 had a dominant (> 70%) chordoid histology (Fig. 1E) and showed extensive brain invasion. The invasive nests at the tumor-brain interface had non-chordoid morphology (Fig. 1F). Mitotic index was high and focally reached 12 mitoses/10 HPF (Fig. 1G). Frequent microscopic necrosis and large geographic necrosis (Fig. 1H) were present, as well as small cell change, hypercellularity, and prominent nucleoli (Fig. 1G). This patient had multiple recurrences and died from progressive disease 88 months after initial resection, despite multiple rounds of radiation therapy and additional surgeries. The three recurrence resection specimens showed atypical meningioma morphology without any chordoid features. The tumor from patient 12 had a mitotic index of 5/10 HPF and areas of hypercellularity with sheeted architecture, but showed no brain invasion, small cell change, prominent nucleoli, or necrosis. This patient had no evidence of recurrence at 38 months of follow up.

**Fig. 2:**
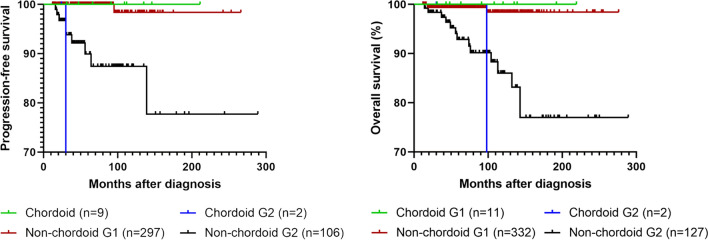
Kaplan–Meier survival analyses of progression free survival and overall survival of chordoid meningiomas compared to non-chordoid meningiomas

Kaplan–Meier survival analyses were performed on patients with > 12 months of brain imaging follow up for progression free survival (PFS) and any follow up for overall survival (OS), to compare the chordoid cohort with non-chordoid cohort (Fig. 2). PFS of chordoid, otherwise grade 1 meningiomas was comparable to non-chordoid WHO grade 1 meningioma (*p* = 0.75, Log-rank test), and significantly better than chordoid WHO grade 2 meningiomas (*p* = 0.019, Log-rank test). Similarly, OS of chordoid, otherwise grade 1 meningioma was comparable to non-chordoid WHO grade 1 meningioma (*p* = 0.71, Log-rank test), and significantly better than chordoid WHO grade 2 meningioma (p = 0.008, Log-rank test).

### Whole genome DNA methylation analysis

DNA methylation analysis of 11 cases of chordoid meningiomas all classified as meningioma by the German Cancer Research Center (DKFZ) meningioma methylation classifier[25], v2.4 (https://www.molecularneuropathology.org/mnp/classifier/5). Of those, nine matched to the benign methylation class (MC) family Ben-2, one matched to the MC family Inter-A, and the only recurrent case matched to malignant family MC Mal (Table 1). None of the cases had MGMT promoter methylation.

**Fig. 3:**
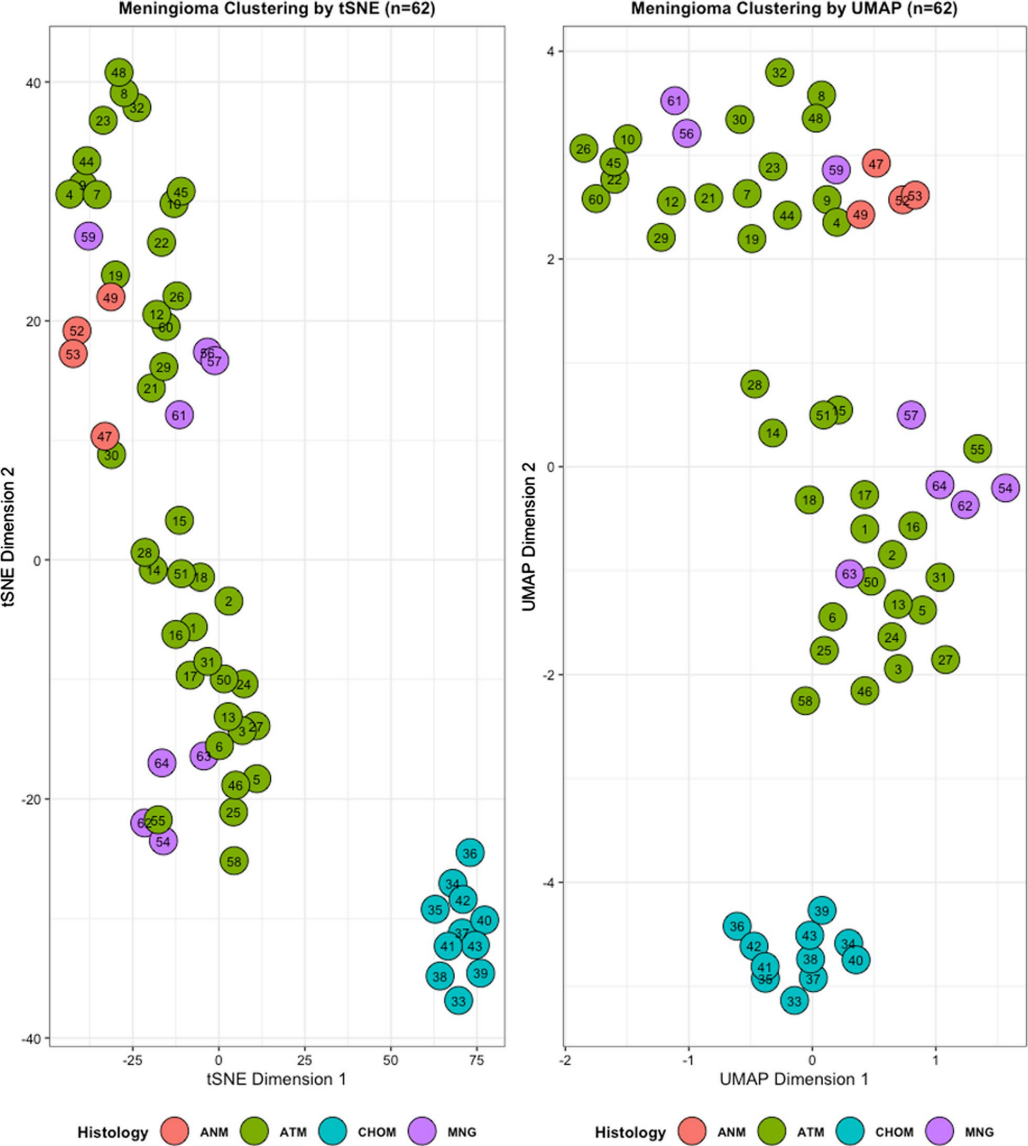
Unsupervised clustering of epigenetic markers from chordoid meningioma samples reveals distinct subtypes. DNA methylation from 62 patient samples was analyzed using the EPIC array; the resulting methylation matrix was then passed through two commonly used dimensionality reduction algorithms, tSNE (left) and UMAP (right). Samples are annotated by histological classification. ATM: atypical meningioma. ANA: anaplastic meningioma. CHOM: chordoid meningioma. MNG: meningothelial and transitional meningiomas

**Fig. 4:**
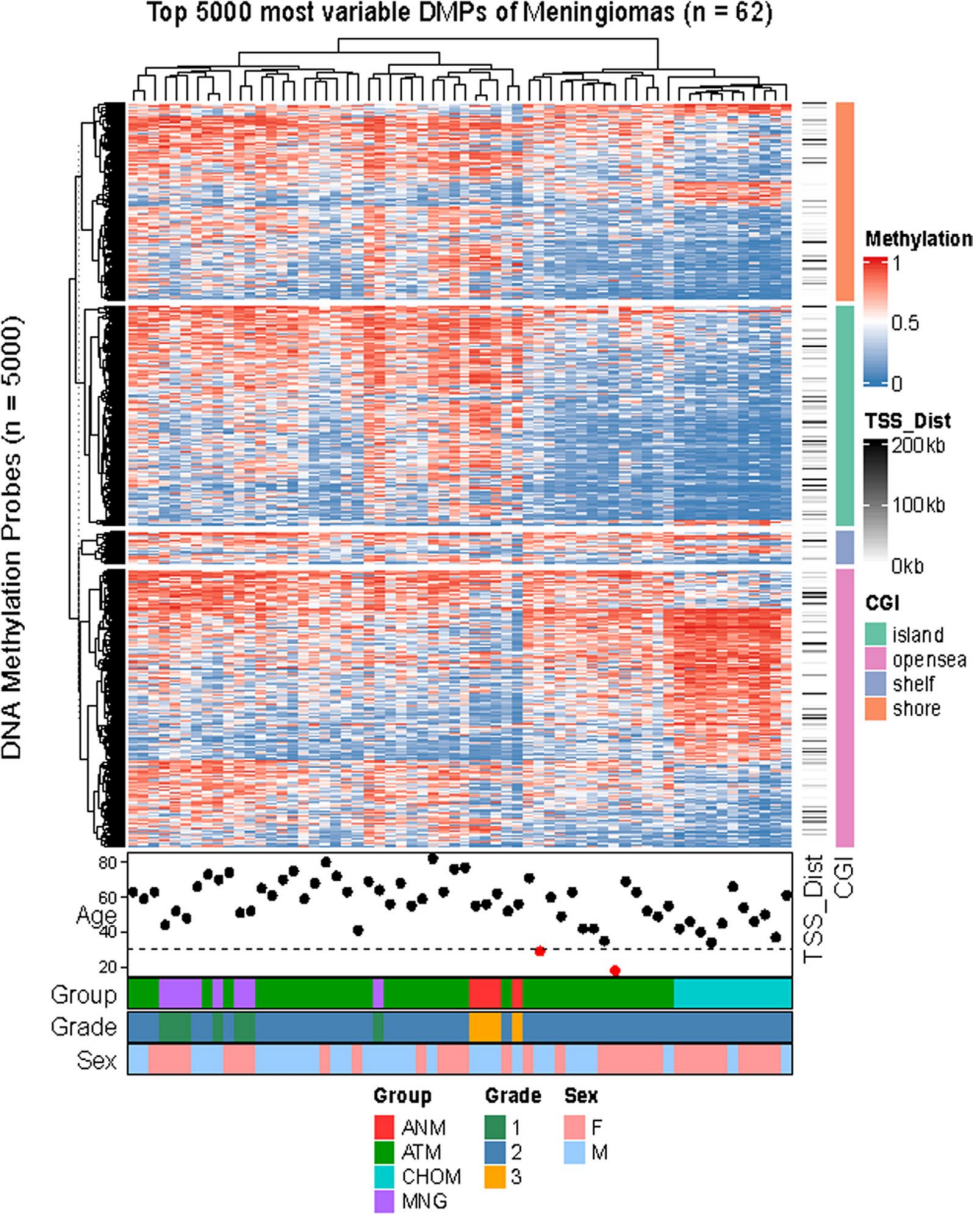
Differentially Methylated Probes (DMPs) analysis of a heatmap of the top 5000 most variable methylated CpGs. Columns represent samples and rows methylation sites; individual cells are color coded by their methylation value. Hierarchical clustering was performed on both axes of the heatmap. The distance of each methylation site from a transcription start site is annotated to the right of the heatmap. Differential methylation highlights the distinct signature of chordoid meningiomas. Meningioma classification is annotated beneath the heatmap: ANM- Anaplastic meningioma, ATM- Atypical meningioma, CHOM- Chordoid meningioma, MNG- meningothelial and transitional

T-distributed stochastic neighbor embedding (t-SNE) analysis and Uniform Manifold Approximation and Projection (UMAP) on 11 chordoid and 51 non-chordoid meningiomas showed that the chordoid meningiomas clustered together, separate from non-chordoid meningiomas (Fig. 3). The 11 chordoid cases also clustered together on the hierarchical clustering analysis of the heatmap of most differentially methylated CpGs (0.05 significant) (Fig. 4).

### Copy number variation analysis

Copy number variations (CNV) of the 11 chordoid meningioma cases are summarized in Tables 1 and 2. The most common copy number variation in chordoid meningioma was loss of 1p (7/11, 64%), which was not significantly different from the control cohort composed mostly of high grade meningiomas (75%, *p* = 0.47). Four chordoid cases had balanced genome with no large losses or gains (all in the Ben-2 MC). Three chordoid meningiomas had 2p loss, which was significantly higher than the non-chordoid control cohort (27% vs 7.2%, *p* = 0.035). All three cases with 2p loss also had 1p loss. Chordoid meningiomas had significantly less 14q loss and 22q loss than the control cohort. Interestingly, 22q loss was only seen in the two chordoid meningiomas with atypical histological features, but in none of the otherwise WHO grade 1 chordoid meningiomas. The case that was classified into Mal MC (Patient 11) showed multiple losses including 1p, 8p, 10, 14, 22q, as well as NF2 truncating mutation and homozygous deletion of CDKN2A/B, alterations commonly seen in atypical and anaplastic meningiomas [3, 10].

**Table 2:** Methylation class and copy number variation of meningiomas

Variables	Overall, N = 62^a^	Chordoid, N = 11^a^	Non-Chordoid, N = 51^a^	*p* value^b^
MC class, n (%)				< 0.001
Benign	16 (26)	9 (82)	7 (14)	
Intermediate	28 (45)	1 (9.1)	27 (53)	
Malignant	18 (29)	1 (9.1)	17 (33)	
1p Loss, n (%)	45 (73)	7 (64)	38 (75)	0.47
2p Loss, n (%)	5 (8.1)	3 (27)	2 (3.9)	0.035
10 Loss, n (%)	15 (24)	1 (9.1)	14 (27)	0.27
14q Loss, n (%)	31 (50)	1 (9.1)	30 (59)	0.003
22q Loss, n (%)	44 (71)	2 (18)	42 (82)	< 0.001

^a^Median (IQR) for frequency (%)

^b^Fisher’s exact test; Person’s Chi-squared test

### Targeted next-generation sequencing

**Fig. 5:**
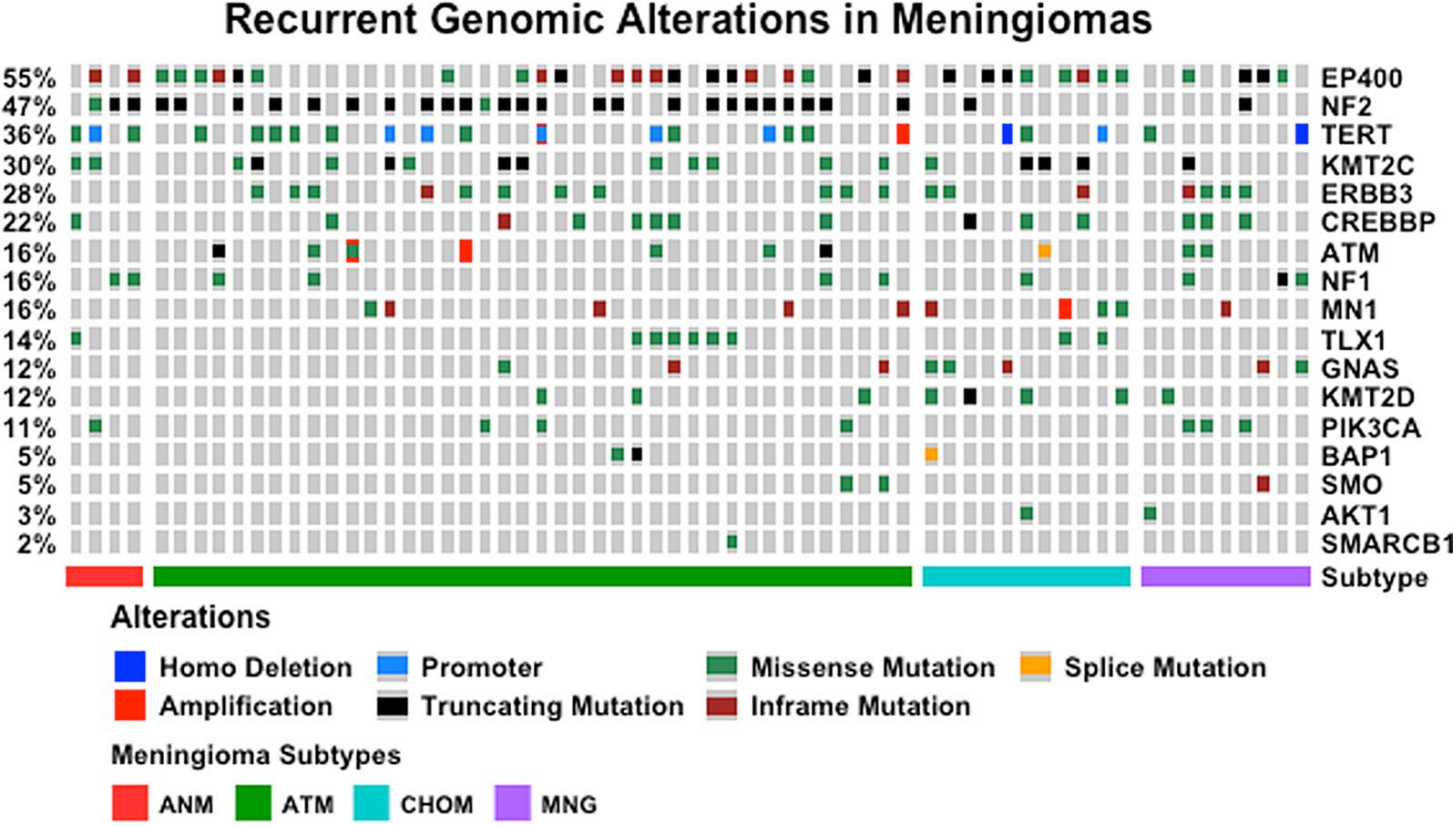
Genomic profiles of chordoid meningiomas. Visualization of recurrent genomic alteration events in Meningiomas by OncoPrint. The samples are clustered by Meningioma type. Alteration percentage per gene is shown on the left side

The genetic changes are summarized in Fig. 5 and Additional file 1: Data S1. Raw annotated variant file including variant allelic frequency (VAF), mean read depth and clinVar annotations are provided in Additional file 2: Data S2. The chordoid meningiomas showed lower rates of mutation than non-chordoid high grade meningiomas, with infrequent *NF2* and *TERT* promoter mutations. Mutations common in non-chordoid low grade meningiomas, such as *AKT1* and *SMO*, were also sparse or absent. Interestingly, chordoid meningiomas appeared to be enriched in chromatin remodeling genes *EP400* (8/11, 73%, mean read depth 291)) KMT2C (4/11, 36%, mean read depth 1715) and KMT2D (4/11, 36%, mean read depth 575) mutations. Those mutations were also present in non-chordoid meningiomas but were less frequent, 49% for *EP400*, 27% for *KMT2C* and 7.2% for *KMT2D*, respectively.

## Discussion

In this study, we demonstrate that the chordoid histology alone may not be predictive of clinical aggressiveness. Rather, aggressive behavior in this chordoid meningioma cohort was associated with concomitant atypical histology and genetic molecular alterations found in conventional atypical meningiomas. Compared to prior studies, we report a unique methylation profile for chordoid meningioma, enrichment of chromatin remodeling gene mutations, and a predominantly benign clinical course in this relatively small cohort.

Demographically, chordoid meningioma accounted for 0.79% of all meningiomas resected at our institution between 1995 and 2018, consistent with previously reported frequency ranging between 0.36 and 1.64% [8, 24, 32]. The mean age at presentation was 46.9, similar as the 45.5 year of age reported in a meta-analysis [1], and nearly a decade younger than the non-chordoid meningiomas (mean 55.0). This feature raised the possibility of a genetic or nongenetic risk that increased the chances of developing meningioma at an earlier age compared to the general population. However, the lack of any significant family history of brain tumors and scarcity of patients with multiple tumors argues against a germline tumor pre-disposition in patients with chordoid meningiomas.

Genetically, previous studies [9, 28] found that chordoid meningiomas had sparce *NF2, TRAF7, KLF4, SMO, AKT1* and *SMARCB1* mutations common to non-chordoid meningioma. In accordance, our study found *NF2* and *AKT1* mutation in one case each, and no *SMO* or *SMARCB1* mutation. *TRAF7* and *KLF4* were not included in our relatively small NGS panel. Our chordoid cohort was relatively enriched in chromatin remodeling genes *EP400*, *KMT2C* and *KMT2D* mutations compared to non-chordoid meningiomas. EP400 (E1A binding protein, 400KD) is the central ATP-hydrolyzing subunit of the TIP60/EP400 complex which utilizes the energy from ATP hydrolysis to reorganize chromatin and gene expression [12]. KMT2C and KMT2D are type 2 lysine methyltransferases that form the core of nuclear regulatory structures known as COMPASS complexes (complex of proteins associating with Set1). KMT2C/D mediates mono- and tri-methylation of histone H3 at lysine 4 (H3K4me1 and H3K4me3) [16]. However, the mutations in *KMT2C/D* in our chordoid meningiomas cohort did not result in diffuse loss of nuclear H3K4me1 or H3K4me3 expression by immunohistochemistry (data not shown). These mutations were not present in every chordoid case and were also seen in non-chordoid meningiomas. Our study also confirms the finding by Sievers et al.[28] that 2p loss was significantly higher in chordoid than non-chordoid meningiomas. However, 2p loss was only present in 27% of our chordoid cases and not associated with aggressive behavior.

In a large genetic study comparing 468 benign and 88 atypical primary meningiomas, Harmanci et al*.* [10] reported that the only somatically mutated gene that was found to be significantly enriched in the atypical cohort was *NF2*, which co-occur with either genomic instability or *SMARCB1* mutations [10]. This finding held true in our chordoid cohort as well. Recurrence was only seen in the one case with *NF2* truncating mutation along with chromosome instability, and atypical histology was associated with 22q loss. Likewise, in Georgescu et al*.* [9], the worst prognostic group (ED2) had 75% cases with *NF2* mutation, 71% with 22q loss. The intermediate prognostic group (ED3) had 12% case with *NF2* mutation, 85% with 22q loss. The benign group (ED1) had 0% *NF2* mutation, and 25% 22q loss. In Sugur et al. [29], 5 of 15 chordoid meningiomas recurred, all of which showed either complete or partial deletion of the *NF2* gene locus; and four of the five cases had deletions of 1p,14q,18p and 22q. Taken together, it appears that *NF2* mutation and chromosome instability can account for majority of recurrences in chordoid meningioma.

Intriguingly, tSNE, UMAP, and hierarchical clustering heatmap analyses of most differentially methylated CpGs did identify a unique methylation profile that separated the chordoid meningioma cohort from the non-chordoid control cohort. We speculate that the chordoid morphology may have an underlining genetic or epigenetic alteration that occurred early in tumorigenesis, which in itself does not portend aggressive behavior. However, gaining of additional molecular features of high-grade meningioma, such as 22q/*NF2* loss, chromosome instability, and homozygous *CDKN2A/B* deletions leads to tumor progression. The variable presence of the latter high grade meningioma molecular features in different chordoid meningioma series may account for the variable recurrence rates in those studies. That been said, this study is limited as a small, retrospective cohort from a single institution, and liable to unintended selection bias. The control cohort is also relatively small and unbalanced, composed predominantly of atypical and anaplastic meningiomas, which may affect hierarchical clustering analyses of methylation profile. More data from a larger dataset in future studies will be necessary for confirmation.

The association between the extent of chordoid component and tumor aggressiveness has been controversial. In a series of 42 chordoid meningiomas, Couce et al. reported that 85.7% of recurrent tumors had primary tumors with > 50% chordoid pattern. Lin et al. [18] reported increased chordoid component in one of two recurrent tumors compared to their primary counterparts. On the other hand, Sadashiva et al. found no correlation between the extent of chordoid component and recurrence in a series of 41 chordoid meningiomas [24], nor did Choy et al. in a large meta-analysis [1]. In our series, none of the tumors with pure chordoid morphology recurred after long term follow up. The only case that did recur had 70% chordoid component in primary tumor and no significant chordoid component in 3 separate recurrence specimens. Atypical features such as invasive tumor nests in brain (Fig. 1F), dura or bone at the periphery of tumor typically had non-chordoid morphology; necrosis often occurred in regions of solid hypercellularity that lacked chordoid matrix (Fig. 1G).

There have been rare, individual case reports of “myxoid meningioma”, which was considered as a rare variant of metaplastic meningioma that generally behaved in a benign fashion [23, 26]. The distinction between chordoid and myxoid meningioma was morphological, emphasizing vacuolated cytoplasm, well demarcated cell borders and polygonal cell shape in chordoid meningioma. No genetic molecular studies have been done on myxoid meningiomas specifically. However, metaplastic meningioma in general were enriched in chromosome 5 gain, and classified into the Ben-3 methylation class [25], which was distinct from our chordoid cohort.

Chordoid meningioma is currently classified as WHO grade 2. However, this designation creates a conundrum in practice for tumors with marked chordoid matrix but otherwise benign histology, which behaved as WHO grade 1 meningioma in our experience. Georgescu et al. and Sievers et al. each proposed a 3-tiered grading system within chordoid meningioma depending either on the presence of different mutations and/or the DNA methylation subclass. In cases where DNA methylation or panel genetic analysis is not available, reporting of presence or absence of any other atypical features is highly recommended.

## Supplementary Information

**Additional file 1**: Detailed clinical and histopathological information on each chordoid meningioma case.

**Additional file 2**: Raw annotated variants detected in chordoid meningiomas.

## Data Availability

The datasets generated and/or analyzed during the current study are available in the GEO repository (GEO accession number: GSE200321).
